# Captive Housing during Water Vole (*Arvicola terrestris*) Reintroduction: Does Short-Term Social Stress Impact on Animal Welfare?

**DOI:** 10.1371/journal.pone.0009791

**Published:** 2010-03-24

**Authors:** Merryl Gelling, Iñigo Montes, Tom P. Moorhouse, David W. Macdonald

**Affiliations:** Wildlife Conservation Research Unit, Department of Zoology, University of Oxford, Oxford, United Kingdom; University of Bristol, United Kingdom

## Abstract

**Background:**

Animals captive bred for reintroduction are often housed under conditions which are not representative of their preferred social structure for at least part of the reintroduction process. Specifically, this is most likely to occur during the final stages of the release programme, whilst being housed during transportation to the release site. The degree of social stress experienced by individuals during this time may negatively impact upon their immunocompetence.

**Methodology/Principal Findings:**

We examined two measure of stress - body weight and Leukocyte Coping Capacity (LCC) - to investigate the effects of group size upon captive-bred water voles destined for release within a reintroduction program. Water voles were housed in laboratory cages containing between one and eight individuals. LCC scores were negatively correlated with group size, suggesting that individuals in larger groups experienced a larger degree of immuno-suppression than did individuals housed in smaller groups or individually. During the course of the study mean body weights increased, in contrast to expectations from a previous study. This was attributed to the individuals sampled being sub-adults and thus growing in length and weight during the course of the investigation.

**Conclusions/Significance:**

The reintroduction process will inevitably cause some stress to the release cohort. However, for water voles we conclude that the stress experienced may be reduced by decreasing group size within captive colony and/or transportation housing practises. These findings are of significance to other species' reintroductions, in highlighting the need to consider life-history strategies when choosing housing systems for animals being maintained in captivity prior to release to the wild. A reduction in stress experienced at the pre-release stage may improve immunocompetence and thus animal welfare and initial survival post-release.

## Introduction

The maintenance of animals in captivity as part of a larger program to ensure a particular species' survival is becoming increasingly commonplace [1]. Within the realms of species reintroduction and translocation, individuals or groups of some species are routinely bred and/or held in captivity prior to being released into the wild. Animals are often housed under conditions which may not be representative of their preferred social structure, and this is particularly likely during transportation to the release site [2]. Although much research has been conducted into social group size and optimal housing conditions in, for example, zoos [3] and for some species groups, specifically primates [4], and felids [3], little attention has focused upon those animals being housed or transported for the purposes of reintroduction or translocation. In many cases housing conditions are defined by convenience for the establishment, rather than animal welfare considerations [5]. It is, however, known from observed changes in cortisol levels that wild animals can become stressed after exposure to captivity for short periods of days or even hours [6], [7].

In this paper, we follow Moberg's [8] definition of stress as being ‘the biological response elicited when an individual perceives a threat to its homeostasis’. Housing animals in artificial conditions can induce a number of innate biological responses which may impact negatively upon individuals, not least due to the restricted movement imposed by a small pen or cage [2]. Overcrowding, or housing of animals in abnormal social groupings, can induce chronic stress from which individuals are unable to retreat [2]. Mugnai *et al* [9] found that social female rabbits kept in colony cages demonstrated an increased incidence of disagreeable social encounters, thus reducing animal welfare standards. Under laboratory conditions, floor space requirements based solely on rodent weight is used for determining housing density [10] but does not differentiate between requirements of different social groups and the additional stress that group composition and size might engender [10], [11]. The social stress inflicted by increased cage density has been shown to compromise growth rates in rats [12] and some mouse strains, but to have the opposite effect in a different mouse strain [13]. Animal welfare standards may therefore be compromised by not optimising housing density for a given species. This may be particularly undesirable in the captive breeding of endangered species [2].

To increase the chances of a ‘successful’ reintroduction, it is desirable to release animals in the best condition possible [14]. It is therefore vital that the well-being of animals held in captivity prior to release is managed with careful consideration of the life-history traits of the species in question, and by the monitoring of a suite of physiological and behavioural indices. Some literature already exists investigating the effects of stress on behaviour of captive animals destined for release (e.g. [15], [16] and on the effects of transport of wild and translocated animals (e.g. [17], [18], emphasising the need for further research into the impact of conditions under which animals are maintained and transported prior to release to the wild.

In this paper we investigate the effects of group size upon captive-bred water voles (*Arvicola terrestris*) destined for release as part of a reintroduction project (see [19], [20]. Water voles are an endangered species in the United Kingdom [21], [22], [23], and reintroductions are likely to be a necessary part of any future species conservation plan (e.g. [23]. A number of institutions are currently involved with captive breeding for release in the UK, and routinely deliver animals for reintroduction in laboratory cages of the type supplied by Big Apple (Big Apple Pet Supply, New York) housing single animals or, more commonly, same-sex groups [20]. Moorhouse *et al* [24] have previously demonstrated that individually-housed water voles in laboratory cages may be physiologically stressed in comparison with individuals housed in larger outdoor enclosures. We wished to ascertain whether there were potential adverse consequences of the impact of group size and composition for the release cohort deriving from pre-release housing conditions.

We used two published measures of stress; body weight [25] and Leukocyte Coping Capacity (LCC) [26], [27], to investigate the effects of water vole group size and composition when housed in laboratory cages. LCC is measured using a challenge–coping approach and provides a quantifiable measure of the stress experienced by an individual by chemically stimulating an immune challenge *in vitro* in a small amount of whole blood using phorbol 12-myristate 13 acetate (PMA). These results are then compared to an animal's basal immune system response challenge [27], [28]. Body weight has been shown to be affected by stress [29], [30] and LCC has been demonstrated to be reflective of stress levels [8], [17], [24], [27], [31]. These techniques measure different parameters within the stress response and thus the use of both is merited.

In this experiment, we hypothesise that animals subjected to known stressors (in this case cage crowding) should have a lower LCC than animals that have not [32], [33], [34]. We test the prediction that water voles will have a lower LCC score when they are housed in larger groups compared with when they are housed in smaller groups or individually.

## Methods

### Study background and husbandry

Eighty-nine captive-bred water voles were obtained from two breeding establishments for release as part of a large-scale reintroduction experiment. Water voles were collected from the captive breeding establishments on 30/04/07 and 02/05/07, respectively, and maintained in captivity pre-release in specialist facilities in the Department of Zoology, University of Oxford. Two reintroductions into sites within the Upper Thames region around Oxford took place on the 11/05/07 and 18/05/07, each comprising half of the captive population. Individuals were therefore maintained in captivity for a maximum of 18 days following transportation to Oxford. This period allowed for pre-release health checks to derive baseline measurements for levels of physiological stress and disease screening (details to be reported elsewhere).

Water voles had been bred in outdoor enclosures measuring 1.8×1.2×1.2m late during the previous breeding season (September 2006 onwards) and maintained throughout the winter. One week before transportation to Oxford the voles were captured by hand from their breeding pens, and split into smaller, same-sex groups in standard lab cages, as per standard practice for captive-bred water voles destined for reintroduction programs. Lab cages of two sizes were used: large (34×56.5×18.5 cm) and small (25.5×42.5×20 cm); these were supplied by the breeding establishments and both establishments used both cage sizes. The cage size into which individuals were placed was arbitrarily determined according to the number of individuals of each sex captured in each breeding pen; all individuals of one sex were moved onto one lab cage. Numbers of individuals in a given lab cage ranged from one to eight (mean = 2.25, s.d. = 1.03). All animals were kept in the same cages until the reintroduction took place, excluding those cages containing large numbers of individuals (five plus) which were further separated out in Oxford for welfare and logistical reasons – see Study design for details.

During captivity (both whilst housed in breeding pens and lab cages) each water vole was fed ¼ fresh apple daily with chopped vegetables and dried rodent food (Russell Rabbit, Supreme Petfoods Ltd., Waterlooville), with access to water provided *ad libitum* via standard lab water bottles which were replenished daily. Cages containing multiple animals had the equivalent amount of food supplied per individual. Cages were cleaned weekly on a rotational basis, organised to ensure that cleaning did not occur within four days either side of screening to prevent any potential impact on stress levels. Where possible, cage cleaning occurred whilst the animals were being screened. Sawdust and hay were used for bedding; the hay was re-used but supplemented where necessary to maintain olfactory familiarity. Whilst housed in lab cages environmental conditions were set to mimic natural conditions for the time of year: 15 hours of light and nine hours of darkness, with an ambient temperature of 18°C.

### Study design

We wished to examine the influence of group size upon measures of stress. The study design was necessarily observational in that, due to the requirements of the subsequent reintroduction, we were unable explicitly to manipulate group sizes in each cage in response to a predetermined design, and no controls were possible. The group sizes in the study therefore reflected the prevailing number of voles in a given cage at the time of sampling.

The water voles arrived at Oxford in 31 separate laboratory cages. Animals in large groups (>five voles per cage) were further separated into another six laboratory cages to aid separation of blood lines across reintroduction sites. For the individuals being sampled immediately, the number of voles in their cage was recorded as the number of individuals in the original cage. The re-housed portion was sampled a minimum of 10 days later to allow ample time for acclimatisation to the new group size [35], and the number of voles in their cage was recorded as the re-housed number of individuals.

The data for this study comprise LCC and weight measurements of animals sampled over 18 days of captivity pre-release from a total of 37 cages (Table 1). Although all captive animals were sampled for further work (data to be published separately), to control for non-independence of individuals within laboratory cages, and to remove potential effects of the time that individuals waited in the holding container prior to sampling (see below), all analyses in the present study were conducted using data only from the first individual sampled from each cage (37 individuals; see Table 1).

**Table 1:** The sampling design of the study.

Cage size	Number in cage	Number of cages
Large	1	2
Small	1	2
Large	2	7
Small	2	7
Large	3	7
Small	3	2
Large	4	6
Large	5	1
Large	6	2
Large	8	1

The table presents the number of cages (37 in total) of each type, defined by cage size and number of occupants that were sampled. Only data from the first individual sampled from each cage were analysed, so the number of cages also represents the number of individuals sampled within each level of “Number in cage”.

We used the number of voles in each cage as the measure of group size, as opposed to measures of density such as units of space available per unit of body weight. This was because water vole weights can vary greatly, and with cages of two different sizes, available space in each cage alone may not have adequately represented the water voles' experience of their housing conditions (for example, two 300 g adult male water voles in a given cage may have a similar social experience to two 250 g adult male water voles but the metric of area available per gram would be very different). Effects of cage area were accounted for by including them as a separate variable in the analysis (see Data analysis, below).

### Sampling methods

Sampling was conducted on a cage-by-cage basis. All individuals in a single cage were moved into a large holding container (1×1.5×1 m) with additional food and refuges (a clean, inverted laboratory cage with bedding, and cardboard tubes with one closed end). A small amount of bedding from the original cage was also added to maintain olfactory familiarity. The voles had routinely been moved into a similar container for cage-cleaning purposes both whilst at the captive breeding establishments and when in Oxford, and were therefore familiar with this procedure. The original cage was then cleaned, as per normal husbandry practice, and re-provisioned with food in readiness to house the animals post-sampling.

Sampling of the release cohort took place over nine separate days within an 18 day period. All animals destined for each reintroduction site were sampled at least three days prior to the reintroduction taking place, to allow the individual time to recover before being released. The sampling programme was carefully arranged to ensure that equal sex ratios were sampled on any given day and that the distribution of sampling effort was equally distributed between cages of different sizes containing different numbers to exclude the possibility of temporal bias. The cage to be sampled in any one session was randomly selected from all of those available which met the requirements of the session.

A given day of sampling comprised multiple two-hour sampling sessions. Up to four water voles were sampled in a single sampling session. Once in the holding container, animals were removed individually by encouraging them into a close-ended cardboard tube, following the normal handling protocol for this species. Individuals were anaesthetised directly in this tube using gaseous isoflourane (Isocare, Animalcare Ltd, York, UK: [36] - carried on 99.5% medical oxygen dispensed at a rate of 5% isoflourane delivered at 4 l min^−1^ directly into the tube. Animals were removed from the tube once they had lost their righting reflex (within 15 seconds) and were maintained on isoflourane (2%) delivered at 2 l min^−1^ via a face mask. The effects of the anaesthetic isoflourane on the dependent measures within this study have not been investigated, but are unlikely to have any significant effect. Altholtz *et al* [37] found that the use of isoflourane throughout a repeated measure investigation in comparison to a CO_2_∶O_2_ anaesthesia regime produced a lower overall stress response, measured via serum corticosteroid, although the initial stress response was higher in those animals anaesthetised using isoflourane than CO_2_∶O_2_ mix. It is likely that there is an element of stress experience by individuals within this experimental regime [38] but this was minimised as far as possible by utilising a handling regime familiar to the animals undergoing normal husbandry practices. In addition, each individual underwent the same procedure, thereby standardising the procedure and therefore measuring the background levels of stress beyond that caused by the experimental treatment alone.

### Blood sampling and measurement of Leukocyte Coping Capacity

Once the individual was anaesthetised, their weight was measured to the nearest gram using digital scales, and head-body length measured to the nearest millimetre. Blood was taken by tail venepuncture using a 23G needle. Tail hair was trimmed along the lateral vein, and the area thoroughly swabbed with ethanol to remove bacterial contaminants. Thirty µl of whole blood was collected from each animal, through the needle, directly into a heparin-coated 75 µm glass capillary tube (See [27] for details). A multivette (Multivette 600 K3E, Starstedt, Germany) was then attached to the needle to collect a blood sample for health screening purposes (details to be reported elsewhere).

Ten µl of heparinised whole blood was transferred into a silicon anti-reflective tube (Lumivial E G and G Berthold Germany) and challenged with 10µl 10^−4^ mol l^−1^ Phorbol 12-Myristate 13-Acetate (PMA; Sigma P8139) in the presence of luminol (90µl of 10^−4^ mol l^−1^ luminol (5-amino-2,3-dihydrophthalzine; Sigma A8511) diluted in phosphate buffer pH 7.2.

Leukocyte Coping Capacity (LCC) was measured as the whole blood chemiluminescence response to PMA challenge. The basal chemiluminescence of blood that had not been stimulated by PMA was also measured; this acts as a baseline or control with which to compare the individual's LCC. PMA is regularly used in research on various mammalian species to provoke a leukocyte response [39]. Animals with a higher LCC have a greater potential to produce a respiratory burst and, from a physiological viewpoint, are more readily able to respond to bacterial challenge after being stressed [27]. After a putatively stressful experience, the capacity of the individual's leukocytes to produce a quantifiable immune response, also known as the respiratory burst, is measured *in vitro*. During the respiratory burst leukocytes increase their oxygen uptake in order to produce oxygen free radicals that destroy bacteria (a process reviewed by Gutteridge and Halliwell ([40].

For each sample chemiluminescence was measured by calculating an average over 30 seconds every five minutes in a portable chemiluminometer (Junior LB 9509 E G and G Berthold Germany) for a total of 30 minutes. When not in the chemiluminometer, tubes were incubated at 37°C. LCC is measured in Relative Light Units (RLUs) which are an arbitrary, but internally consistent measurement displayed by a given chemiluminometer.

Total leukocyte (white blood cell) counts were made for each individual using a haematology analyser (Advia 120, Bayer, New York, USA).

### Data analysis

We tested for effects of number of water voles in each cage upon LCC score using multivariate analysis of variance (Minitab ver. 15.1). The dependant variables were LCC score at each five minute time period over the 30 minutes during which LCC scores were measured. Sex was included as an explanatory variable to control for any variation between sexes. LCC scores could potentially be affected by weight, and so this was also included as a covariate. A previous study [24] showed LCC scores and body weights to decrease over the course of the study. We therefore included date of sampling in the models, measured as days since transportation to Oxford. We included cage area as an explanatory variable as this may have had an effect upon LCC scores regardless of the number of occupants. To discount the possibility that the observed LCC scores are a function of the number of leukocytes as opposed to the leukocyte activation levels, leukocyte count was also included in the model [27].

We tested for effects of the number of voles in each cage upon body weight using General Linear Models (GLM; Minitab ver. 15.1), including sex and day of sampling as explanatory variables to control for variation between sexes and any temporal variation in weight during this period.

LCC scores were log transformed to meet assumptions of normality and heteroscedasicity. Body weights and leukocyte counts were left non-transformed. In these forms the variables did not depart from the assumptions of the tests.

### Ethics statement

This work was part of a larger study on the reintroduction of water voles, approved by the Zoology Ethical Review Committee, a subsidiary of Oxford Universities Animal Care and Ethical Review (ACER) Committee. Work was carried out under Home Office Licence 30/2318.

## Results

### Effects of group size on LCC

LCC scores were negatively correlated with the number of water voles in each cage (MANOVA F_7,21_ = 2.506, P = 0.049). Back-transformed marginal mean LCC scores for the lowest and highest number of voles per cage were 140.0 and 83.1 Relative Light Units (RLU), respectively.

No significant effects of sex (MANOVA F_7,21_ = 0.737, P = 0.644), body weight (MANOVA F_7,21_ = 1.469, P = 0.232), cage area (MANOVA F_7,21_ = 1.026, P = 0.443), day of sampling (MANOVA F_7,21_ = 0.395, P = 0.894) or leukocyte count (MANOVA F_7,21_ = 1.150, P = 0.371) upon LCC were indicated.

### Body weight analysis

There was no evidence that the number of voles per cage significantly affected body weight (GLM effect of voles per cage F_1,36_ = 0.09, P = 0.771). During the course of captivity at Oxford (18 days in total), the mean weight of water voles increased (GLM effect of day F_1,36_ = 16.13, P<0.001). Back transformed marginal means indicate that mean weight increased by 59.5 g over the 18 day period during which sampling took place. Males were heavier than females throughout (GLM effect of sex, F_1,36_ = 13.16, p = 0.001), with mean weights of 184.7 g and 160.3 g, respectively. Both sexes increased in length throughout the study (GLM effect of day F_1,37_ = 4.85, P = 0.035 in a model testing for the effects of sex and day of sampling upon length).

## Discussion

In this study there were measurable differences in LCC score of the captive population, which correlated negatively with the number of water voles in each laboratory cage. Whilst the mechanisms that cause changes in leukocyte activity as a result of a stressor are not clearly understood at present, they are thought to involve secretion of corticosteroids and the involvement of several cytokines [41], [42]. Lowered LCC scores suggest immuno-suppression and can be an indicator of physiological stress in mammals [8], [17], [24], [27], [31]. Our LCC results therefore indicate that water voles housed in larger groups were more stressed than water voles housed in cages containing fewer animals. Chronic stress is known to adversely affect growth rates [2] and general health [43] and therefore these findings should impact on future recommendations for husbandry practice, by encouraging breeding establishments, and consultants translocating animals, to routinely house captive water voles in smaller groups, or individually, even if only for short periods of time.

For the analyses we used data from only the first individual for which LCC was measured in each cage. In doing so we controlled for pseudoreplication between individuals from a given cage, and also removed the possibility that the amount of time that individuals waited in the holding container could have introduced a source of error by affecting LCC scores (for instance if individuals had become increasingly stressed following removal from their original cage). It is highly unlikely that the observed negative relationship between LCC and the number of individuals per cage could have arisen due to our use of data only from the first measured animal: for this to be the case, increasing physiological stress would have to increase the probability of capture within cages containing multiple individuals. Moreover, re-running the analysis using one individual randomly sampled from each cage yielded the same results (data not shown) as the presented analysis.

This study only investigates one aspect of potential stress arising from one facet of a reintroduction program. LCC is measured in arbitrary units; whilst the measure allows us to demonstrate an association between levels of overcrowding and this measure of physiological stress, we are unable to make any inference concerning the magnitude of the effect in terms of the levels of stress experienced by the study animals. However, housing is an area which is amenable to simple mitigation in future reintroduction programmes to reduce the additional impact of overcrowding in the face of the many other stressors which might be unavoidable (e.g. transport, Montes et al. 2004). We found no relationship between LCC and leukocyte count, however, both McLaren *et al* [27] and Honess *et al* [44] have shown immune cell activity to be independent of cell numbers.

Body weights were not affected by the group size of water voles in each cage despite a previous study demonstrating that changes in housing conditions (from external enclosures into indoor, singly-housed lab-cages) correlated with weight loss in water voles [24]. A key difference between the water vole populations in these studies is that animals in the present study were born in late autumn and would not be expected to have reached full adult weight at the time of sampling (May) whereas the previous study was conducted later in the year (July/August) when the animals were full adults. The increase in weights of individuals sampled over the course of this investigation is attributable to the water voles growing in size, indicated by the concurrent increase in individual lengths over the sampling period. In this case, therefore, we were unable quantifiably to measure effects of stress upon recorded body weight because such effects would manifest in differences in growth rate rather than absolute measures of weight or weight∶length ratio. Due to the design of the sampling (one sample per individual), comparison of growth rates between individuals over this period was not possible.

Our study suggests that the individuals housed in large groups sizes may have been physiologically stressed compared with less-densely housed individuals. Moorhouse *et al* [24] demonstrated that long-term housing of water voles in single laboratory cages correlated with elevated levels of physiological stress and lower weights, compared with individuals housed in outdoor enclosures. LCC values are not directly comparable between separate luminometers and body weights may have varied seasonally, making direct comparison between the present study and Moorhouse *et al's* [24] study impossible. It is plausible, however, that because multiple individuals housed in laboratory cages had lower LCC scores than singly housed individuals, the lower mean weights in the present study may indicate that the multiply-housed water voles were more physiologically stressed than those reported in Moorhouse *et al* [24].

Our LCC results have clear implications for pre-release housing, specifically for water voles, but potentially also for other species undergoing translocation or reintroduction. Water voles destined for conservation restoration programmes are bred in large outdoor pens. It is logistically unavoidable that transfer to the reintroduction site requires housing in laboratory cages, or equivalent, for an intermediate period. The data from this study, and that of Moorhouse *et al* [24] indicate that this time should, however, be minimised as far as possible. Whilst it is expedient to house water voles in groups in laboratory cages prior to reintroduction for ease of both transport and release, the considerations of ensuring that individuals are in the best physical condition possible for release, and the associated ethical considerations of ensuring good standards of animal husbandry and welfare, suggest that housing water voles in single units should be a standard practice. The reintroduction process will undoubtedly, by its very nature, be the cause of some stress to the individuals involved; individuals must be bred, prior to being transported and ultimately released into a novel environment, all of which requires handling to some extent, regardless of the species in question. Therefore seeking out causes of stress and ameliorating them wherever possible to improve animal welfare would be expected to benefit the overall success of the reintroduction process.

Whilst the current study focuses upon the use of the LCC technique for monitoring stress and animal welfare of individuals undergoing reintroduction, it also has potential to become a valuable tool within laboratory situations by providing an individual measure for group-housed animals. Much work has been conducted on housing density of laboratory rodents (e.g. [10], [11], [35] to identify appropriate stocking densities for different species; the use of an additional tool to predict immunocompetence of individuals will add a further dimension, particularly where routine sampling is conducted and thus the additional blood requirements for this technique will not become an additional stressor.

### Limitations

This study was necessarily limited to measuring physiological indices of stress. Due to the overarching requirements of the reintroduction programme we were unable to manipulate numbers in a given laboratory cage or to take measurements from control animals. Similarly, we were unable to support the physiological measurements taken with more standard measures of faecal corticosteroid or behavioural observations. In the former case this was due to difficulties associated with directly attributing faeces to a given individual. In the latter case the difficulties related to the cryptic nature of water voles, and a desire to have as little presence in the animal housing as possible pre-reintroduction to minimise that potential source of stress.

The lack of a formal experimental design does not invalidate the central finding of this study that individuals housed in larger groups were more physiologically stressed. Similarly, the majority of studies of animal stress concentrate on measuring only one or two aspects of the stress response, and the absence of behavioural information, whilst clearly a limitation, also does not invalidate our results.

Future assessment of the LCC technique in conjunction with other measures of stress, including corticosteroid measurements or behavioural observations may give increased confidence in the ability of this technique to identify patterns of stress and coping in free-living mammals.
